# SUPREME-HN: a retrospective biomarker study assessing the prognostic value of PD-L1 expression in patients with recurrent and/or metastatic squamous cell carcinoma of the head and neck

**DOI:** 10.1186/s12967-019-02182-1

**Published:** 2019-12-26

**Authors:** Sara I. Pai, Ezra E. W. Cohen, Derrick Lin, George Fountzilas, Edward S. Kim, Holger Mehlhorn, Neus Baste, Daniel Clayburgh, Loren Lipworth, Carlo Resteghini, Nawar Shara, Takashi Fujii, Jun Zhang, Michael Stokes, Huifen Wang, Philip Twumasi-Ankrah, Sophie Wildsmith, Asud Khaliq, Giovanni Melillo, Norah Shire

**Affiliations:** 1 Massachusetts General Hospital Cancer Center, Harvard Medical School, 55 Fruit Street, GRJ 9-904G, Boston, MA 02114 USA; 2 UC San Diego Health System, Moores Cancer Center, La Jolla, CA USA; 3 Massachusetts Eye and Ear, Boston, MA USA; 4 Aristotle University of Thessaloniki, Thessaloniki, Greece; 5 Levine Cancer Institute, Atrium Health, Charlotte, NC USA; 6 Universitaetsklinikum Leipzig, Klinik und Poliklinik fur HNO-Heilkunde, Leipzig, Germany; 7 Department of Oncology, Hospital Universitari Vall d’Hebron & Vall Hebron Institute of Oncology (VHIO), Barcelona, Spain; 8 Oregon Health & Science University, Portland, OR USA; 9 Vanderbilt University Medical Center, Nashville, TN USA; 10 Fondazione IRCCS Istituto Nazionale dei Tumori, Milan, Italy; 11 MedStar Health Research Institute, Hyattsville, MD USA; 12 Osaka International Cancer Institute, Osaka, Japan; 13 Baylor College of Medicine, Houston, TX USA; 14 Evidera, Lexington, MA USA; 15 AstraZeneca, Gaithersburg, MD USA; 16 AstraZeneca, Cambridge, UK

**Keywords:** Biomarker, Head and neck squamous cell carcinoma, Immuno-oncology, PD-L1, Programmed cell death ligand-1, Prognosis

## Abstract

**Background:**

Programmed cell death ligand-1 (PD-L1) expression on tumor cells (TCs) is associated with improved survival in patients with head and neck squamous cell carcinoma (HNSCC) treated with immunotherapy, although its role as a prognostic factor is controversial. This study investigates whether tumoral expression of PD-L1 is a prognostic marker in patients with recurrent and/or metastatic (R/M) HNSCC treated with standard chemotherapy.

**Methods:**

This retrospective, multicenter, noninterventional study assessed PD-L1 expression on archival R/M HNSCC tissue samples using the VENTANA PD-L1 (SP263) Assay. PD-L1 high was defined as PD-L1 staining of ≥ 25% TC, with exploratory scoring at TC ≥ 10% and TC ≥ 50%. The primary objective of this study was to estimate the prognostic value of PD-L1 status in terms of overall survival (OS) in patients with R/M HNSCC.

**Results:**

412 patients (median age, 62.0 years; 79.9% male; 88.2% Caucasian) were included from 19 sites in seven countries. 132 patients (32.0%) had TC ≥ 25% PD-L1 expression; 199 patients (48.3%) and 85 patients (20.6%) had TC ≥ 10% and ≥ 50%, respectively. OS did not differ significantly across PD-L1 expression (at TC ≥ 25% cutoff median OS: 8.2 months vs TC < 25%, 10.1 months, *P *= 0.55) or the ≥ 10% and ≥ 50% cutoffs (at TC ≥ 10%, median OS: 9.6 months vs TC < 10%, 9.4 months, *P *= 0.32, and at TC ≥ 50%, median OS 7.9 vs TC < 50%, 10.0 months, *P *= 0.39, respectively).

**Conclusions:**

PD-L1 expression, assessed using the VENTANA PD-L1 (SP263) Assay, was not prognostic of OS in patients with R/M HNSCC treated with standard of care chemotherapies.

*Trial registration* ClinicalTrials.gov, NCT02543476. Registered September 4, 2015.

## Background

Approximately 60% of patients with head and neck squamous cell carcinoma (HNSCC) are diagnosed with locally advanced disease, which has a 5-year overall survival (OS) rate of approximately 30% [1]. Most patients with HNSCC will eventually experience either local or distant recurrence [2], while approximately 10% of patients with HNSCC will initially present with metastatic disease [3]. Patients with recurrent and/or metastatic (R/M) HNSCC have historically had a poor prognosis [4]. Traditional standard of care for first-line therapy in patients with R/M HNSCC is platinum-based chemotherapy plus cetuximab and 5-fluorouracil [5, 6], yielding a median OS of approximately 10 months [7]. However, this is usually only appropriate for patients who have an acceptable Eastern Cooperative Oncology Group performance status (ECOG PS) and are able to tolerate platinum-based therapy. Patients with R/M HNSCC treated in the second-line setting have a poorer prognosis, with median OS of approximately 4–8 months [3, 8, 9]. Standard therapy in this setting includes single-agent therapies (e.g. methotrexate, docetaxel, or cetuximab) which yield objective response rates (ORRs) of 4–13% in the platinum-refractory setting [3, 8, 9]. More recently, phase III studies have demonstrated that immuno-oncology (IO) agents targeting programmed cell death-1 (PD-1)/programmed cell death ligand-1 (PD-L1) improve OS in both the first-line and second-line settings, with median OS of approximately 13–15 months and 7–8 months, respectively [10–14].

PD-L1 is expressed on antigen-presenting cells and other immune cells (ICs) and is upregulated on HNSCC tumor cells (TCs) [15, 16]. The presence of PD-L1 can be readily detected by immunohistochemistry (IHC) staining [16]. Evidence is building that PD-L1 expression on TCs is associated with improved survival in patients with HNSCC treated with IO agents and yet the role of PD-L1 in outcomes irrespective of treatment (i.e. prognosis) is still unclear, with conflicting reports of PD-L1 as both a negative and positive prognostic factor [17–23]. Therefore, the SUPREME-HN study was conducted to investigate the possible prognostic role PD-L1 expression on TCs has in patients with R/M HNSCC. Here, we describe patient characteristics, OS, and other clinical outcomes related to PD-L1 expression independent of treatment choice [20, 24].

## Methods

### Study design

SUPREME-HN was a retrospective, international, multicenter, noninterventional cohort study based on data derived from established medical records and analysis of archival tumor samples (ClinicalTrials.gov identifier: NCT02543476); for the purposes of this study and for patient selection, the index date was defined as the date of diagnosis of R/M disease not amenable to local therapy.

### Patient population

Patients aged ≥ 18 years with histologically confirmed HNSCC of the oral cavity (tongue, gum, floor of mouth, or other/unspecified part of the mouth), oropharynx, larynx, or hypopharynx were eligible if they had R/M disease not amenable to local therapy with curative intent (surgery, radiation therapy, chemo-radiation). Patients with locally advanced disease amenable to curative local therapy were excluded as were patients who had received prior IO treatment with anti-cytotoxic T-lymphocyte-associated antigen 4, or anti-PD-1, anti-PD-L1, or anti-PD-L2 antibodies for HNSCC.

### Procedures

Archival tumor samples (< 5 years old) were obtained anytime during the disease history from patients who were diagnosed between March 1, 2011 and June 30, 2015. Biopsies or resections from the primary site, lymph node, or distant metastatic sites were provided for analysis as formalin-fixed, paraffin-embedded (FFPE) blocks or sections < 60 days old.

For patients with more than one tissue sample, the most recent sample from the index date was used to determine PD-L1 expression. PD-L1 IHC staining of FFPE tissue samples was performed using the VENTANA PD-L1 (SP263) Assay on the automated Ventana BenchMark ULTRA^®^ platform (Ventana Medical Systems Inc., Tucson, AZ, USA) [25]. PD-L1 expression was scored by pathologists trained by the manufacturer, at an approved central testing laboratory. PD-L1 expression was evaluated for a cutoff of ≥ 25% of TCs with membrane staining for PD-L1 at any intensity (TC ≥ 25%). Exploratory scoring was assessed at TC ≥ 10% and TC ≥ 50%. Patient characteristics were collected including ECOG PS at the index date, smoking habits, alcohol consumption, human papilloma virus (HPV) status, HIV status, and medical history. Tumor characteristics, treatment patterns, and outcome measures were recorded.

### Study endpoints

The study primary endpoint was OS as defined from the date of diagnosis of R/M HNSCC (index date) to time of death due to any causes. OS was reported separately in predefined subgroups based on baseline characteristics (e.g. HPV status, anatomical site of tumor). Secondary endpoints included descriptive analyses of demographics and clinical characteristics distribution with PD-L1 as well as investigator-assessed ORR, duration of response, and progression-free survival (PFS). ORR (complete response + partial response) was based on Response Evaluation Criteria In Solid Tumors (RECIST) v1.1. PFS was assessed from the start of first-line therapy for R/M disease to progression on or after therapy, or death due to any cause (whichever came first), and from the start of second-line therapy to first documented disease progression or death due to any cause (whichever came first).

### Statistical analyses

The sample size to support the primary endpoint was not known a priori and was driven by the number of patients at selected sites with available tissue samples. Based on assumptions of a PD-L1 high prevalence of 25% (TC ≥ 25%), a median OS of 10 months, uniform accrual over 52 months with 10 months’ follow-up from the last patient entering, and exponentially distributed survival times, it was determined post hoc that the study statistics could be powered to the 80% level (two-sided alpha 0.05) to detect a hazard ratio (HR) of 0.7 for PD-L1 high versus low/negative patients for a total of 396 patients and 278 deaths.

Time-to-event endpoints were described using the Kaplan–Meier method. Two-sided 95% confidence intervals (CIs) were provided for the main statistical estimators. OS and PFS were compared between patients with PD-L1 high and low/negative expression for the different cutoffs using a log-rank test at a 5% level of significance. Prognostic value of PD-L1 expression in terms of OS was investigated using a multivariable Cox proportional hazards model where covariates were selected by biological and clinical significance and included age, race, smoking status, alcohol use, metastatic disease, platinum-based therapy, and anatomical site as baseline covariates. Due to the retrospective design of the study, some data were unavailable for collection.

## Results

### Baseline characteristics

Nineteen sites in seven countries screened 513 patients with R/M HNSCC tumors not amenable to local therapy (e.g. surgery or radiation) or at stage IVC between March 1, 2011 and June 30, 2015. The majority of patients (*n* = 213; 51.7%) were from the United States, with the remainder from Greece (*n* = 57; 13.8%), Spain (*n* = 49; 11.9%), Germany (*n* = 35; 8.5%), Italy (*n* = 33; 8.0%), Japan (*n* = 15; 3.6%), and South Korea (*n *=10; 2.4%). Of the 513 patients, 412 met all eligibility criteria and comprised the full analysis set; PD-L1 expression was unknown in 16 (3.9%). The 16 patients with unknown PD-L1 expression were not included in prevalence assessments or outcome assessments unless otherwise stated. Most patients (*n *=400; 97.1%) provided one tissue sample, with 12 patients providing two samples for a total of 424 tissue samples. For patients who provided two samples, PD-L1 expression was determined independently on each sample, and the sample obtained closest to the index date was used to assess PD-L1 expression. Tumor samples were obtained from the primary site in 162/424 cases (38.2%), from recurrent disease in 179/424 cases (42.2%), and from distant sites in 83/424 cases (19.6%).

The median age of patients at or closest to the index date was 62.0 years (range 28.0–93.0; *n *= 411) (Table 1). There were 132 patients (32.0%) who were found to have TC ≥ 25% PD-L1 expression (Table 1) [26–28]. Furthermore, 199 patients (48.3%) and 85 patients (20.6%) had TC ≥ 10% and ≥ 50%, respectively. Among 130 patients with HPV data, 37 were HPV-positive (28.5%). Of the HPV-positive patients, 8 (21.6%) had TC ≥ 25% PD-L1 expression, 17 (45.9%) had TC ≥ 10% PD-L1 expression, and 5 patients (13.5%) had TC ≥ 50% PD-L1 expression.

**Table 1:** Prevalence of PD-L1 expression based on baseline characteristics and HNSCC tumor characteristics

Characteristic, %	*N*^a^	PD-L1 TC ≥ 25% (*n* = 132)	PD-L1 TC < 25% (*n* = 264)
Median age, years (range)		62.0 (38.0–87.0)	62.0 (28.0–93.0)
< 60	167	32.9	67.1
≥ 60	228	33.8	66.2
Sex			
Male	317	30.9	69.1
Female	79	43.0	57.0
Race			
Caucasian	339	32.7	67.3
Black or African American	20	30.0	70.0
Asian	22	50.0	50.0
Region			
United States	205	29.3	70.7
Asia	22	50.0	50.0
Europe	169	36.1	63.9
ECOG PS			
0	73	50.7	49.3
1	87	32.2	67.8
≥ 2	41	26.8	73.2
Tobacco use			
Current	97	26.8	73.2
Former	199	32.2	67.8
Never	78	42.3	57.7
Alcohol consumption			
Current	148	26.4	73.6
Former	123	32.5	67.5
HPV status			
Positive	37	21.6	78.4
Negative	93	25.8	74.2
Timing of tissue sample extraction			
Pre-1st chemotherapy, %	202	30.2	69.8
Post-1st chemotherapy, %	36	25.0	75.0
Type of tumor sample			
Surgical resection	186	34.9	65.1
Surgical biopsy	199	32.2	67.8
Punch biopsy	8	12.5	87.5
Location of tumor sample			
Primary tumor	153	34.0	66.0
From recurrent disease	175	32.6	67.4
From metastatic disease	80	33.8	66.3
Primary tumor site		132	261
Oral cavity	108	43.5	56.5
Oropharynx	61	34.4	65.6
Hypopharynx	21	9.5	90.5
Larynx	99	30.3	69.7
Overlapping lesion	22	22.7	77.3
Stage at index date^b^			
Stage 0–III	17	29.4	70.6
Stage IVA	62	37.1	62.9
Stage IVB	21	23.8	76.2
Stage IVC	230	31.3	68.7
Time from diagnosis to index			
Median, months (range)		11.4 (0.0–475.9)	14.7 (0.0–349.8)
Sites of new metastases post index date			
Local lymph node	89	31.5	68.5
Lung	77	27.3	72.7
Bone	29	37.9	62.1
Distant lymph node	23	34.8	65.2
Liver	23	30.4	69.6
Skin/soft tissue	21	42.9	57.1
Head and neck	11	27.3	72.7
Pleura	9	44.4	55.6

^a^Patients with PD-L1 result *N *= 396

^b^Index date is defined as date of diagnosis of R/M HNSCC not amenable to local therapy

*ECOG PS* Eastern Cooperative Oncology Group performance status, *HNSCC* head and neck squamous cell carcinoma*, HPV* human papilloma virus, *mo* months, *PD*-*L1* programmed cell death-ligand 1, *R/*M recurrent and/or metastatic, *TC* tumor cell

At TC ≥ 25%, the PD-L1 prevalence was higher among females (43.0% vs 30.9% for males), Asians (50.0% vs 32.7% and 30.0% for Caucasians and Black/African Americans, respectively), ECOG PS 0 (50.7% vs 32.2% and 26.8% for 1 and ≥ 2, respectively), and never smokers (42.3% vs 26.8% and 32.2% for current and former smokers, respectively) (Table 1). PD-L1 prevalence decreased with increasing ECOG PS values and was highest in never smokers (compared with current and former smokers) and former alcohol users (vs current).

### HNSCC tumor characteristics

The most common sites from which tumor samples were collected were oral cavity (35.0%; *n *= 143), larynx (33.5%; *n *= 137), and oropharynx (22.2%; *n *= 91). Oral cavity tumors (43.5%) showed the highest prevalence of PD-L1 expression (TC ≥ 25%), while the hypopharynx tumors were most often associated with PD-L1 < 25% (90.5%) (Table 1).

The prevalence of PD-L1 expression TC ≥ 25% was similar irrespective of whether the sample was collected from the primary tumor (34.0%), or recurrent (32.6%) or metastatic (33.8%) sites. There was also no difference in prevalence regarding the type of tumor sample used (34.9% in surgical resection vs 32.2% for surgical biopsy) (Table 1).

### Treatment history

Among the total cohort of 412 patients, 238 patients (57.8%) received first-line chemotherapy and 84 patients (20.4%) received additional second-line chemotherapy after the index date (Table 2). A limited number of patients received subsequent lines of chemotherapy (*n* = 42; 10.2%). First-line chemotherapy was administered to 52.3% of patients in the PD-L1 TC ≥ 25% group and 60.2% in the PD-L1 TC < 25% group. Approximately 30% of patients underwent palliative surgical interventions and another ~ 30% underwent radiotherapy. The most common first-line targeted therapy was cetuximab (49.6%), and chemotherapy treatments were cisplatin (44.7%), 5-fluorouracil (36.5%), carboplatin (31.6%), paclitaxel (25.2%), and docetaxel (16.2%) (Table 2). The rates of prior first-line treatment with cetuximab and platinum-based therapy were similar for patients in either PD-L1 cohort. The most common second-line targeted therapy was cetuximab (33.3%), and chemotherapy treatments included paclitaxel (27.8%), carboplatin (22.2%), docetaxel (20.0%), and 5-fluorouracil (11.1%), again with no differences between PD-L1 expression cohorts (Table 2).

**Table 2:** Treatment history

Treatment history, *n* (%)	PD-L1 TC ≥ 25% (*n* = 132^a^)	PD-L1 TC < 25% (*n *= 264^a^)	Total (*N* = 412)
Palliative surgical interventions	44 (33.3)	74 (28.0)	123 (29.9)
Radiotherapy	43 (32.6)	63 (23.9)	113 (27.4)
Chemoradiation therapy	1 (0.8)	0 (0.0)	1 (0.2)
Line of chemotherapy, *n*	132	264	412
1st	69 (52.3)	159 (60.2)	238 (57.8)
2nd	24 (18.2)	55 (20.8)	84 (20.4)
≥ 3rd	8 (6.1)	28 (10.6)	42 (10.2)
Type of first-line chemotherapy, *n*	77	177	266
Cetuximab	38 (49.4)	91 (51.4)	132 (49.6)
Cisplatin	41 (53.2)	71 (40.1)	119 (44.7)
Carboplatin	15 (19.5)	64 (36.2)	84 (31.6)
Paclitaxel	10 (13.0)	56 (31.6)	67 (25.2)
Docetaxel	18 (23.4)	22 (12.4)	43 (16.2)
5-Fluorouracil	35 (45.5)	56 (31.6)	97 (36.5)
Type of second-line chemotherapy	25	60	90
Cetuximab	8 (32.0)	21 (35.0)	30 (33.3)
Cisplatin	1 (4.0)	4 (6.7)	5 (5.6)
Carboplatin	6 (24.0)	13 (21.7)	20 (22.2)
Paclitaxel	5 (20.0)	20 (33.3)	25 (27.8)
Docetaxel	7 (28.0)	10 (16.7)	18 (20.0)
5-Fluorouracil	5 (20.0)	5 (8.3)	10 (11.1)

^a^Patients with PD-L1 result *N *= 396

*PD*-*L1* programmed cell death-ligand 1, *TC* tumor cell

### Treatment outcomes

**Fig. 1:**
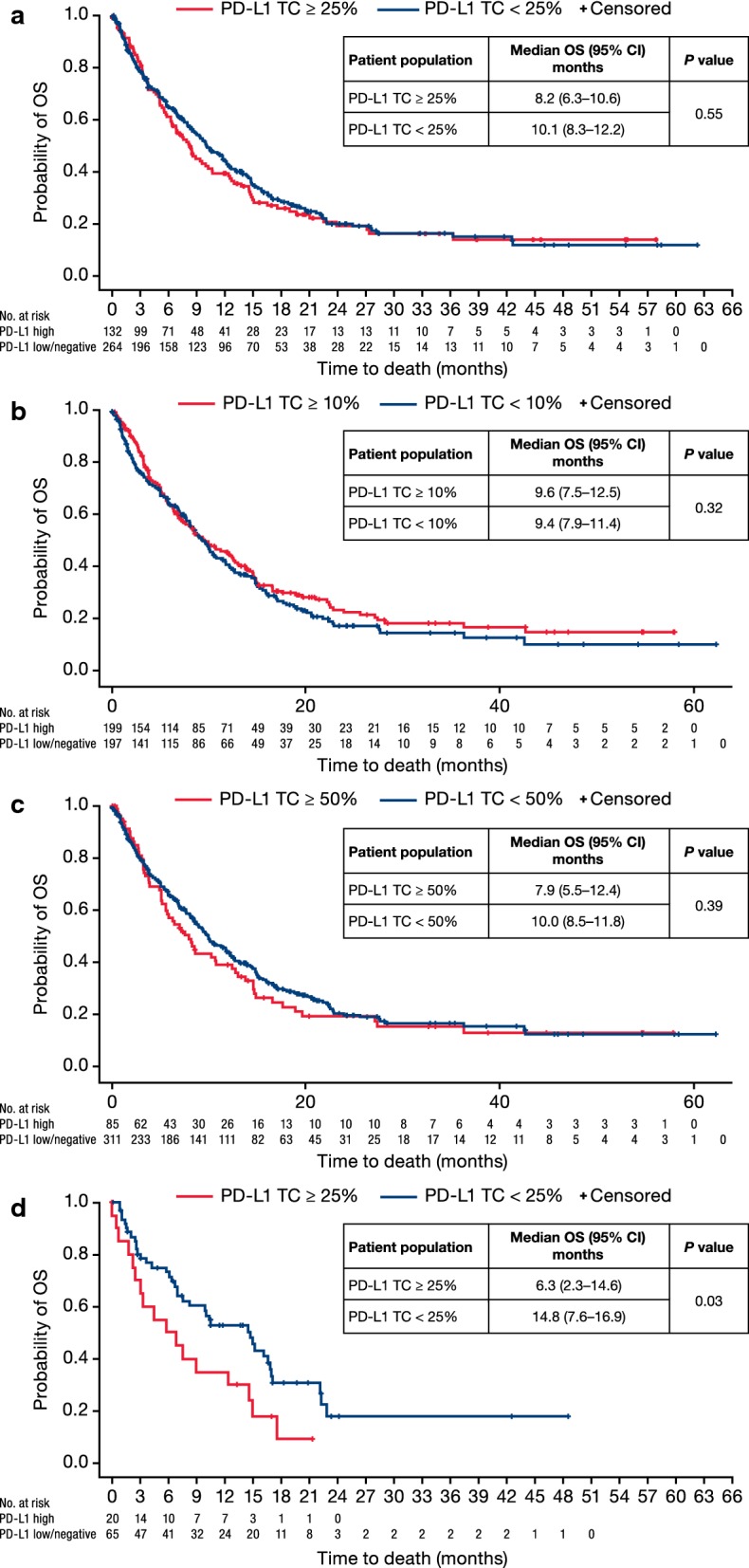
Overall survival (OS) by PD-L1 expression^a^. ^a^Patients with PD-L1 result *n* = 396: **a** TC ≥ 25%, **b** TC ≥ 10%, and **c** TC ≥ 50%; **d** oropharynx anatomical sub-site (*n* = 91) by PD-L1 status

A total of 290 (70.4%) patients died during the study period. Median OS from the index date of R/M disease was 9.6 months (95% CI 8.3–10.8). Among the patients with known PD-L1 expression, OS did not differ significantly for PD-L1 TC ≥ 25% versus TC < 25% (median 8.2 vs 10.1 months, *P *= 0.55; Fig. 1a). This was also true for PD-L1 expression cutoffs of TC ≥ 10% versus TC < 10% (median 9.6 vs 9.4 months, *P* = 0.32; Fig. 1b) and TC ≥ 50% versus TC < 50% (median 7.9 vs 10.0 months, *P* = 0.39; Fig. 1c). Among the 130 patients with available HPV status, median OS was 10 months (95% CI 5.1–16.9) in patients with HPV-positive status and 8.3 months (95% CI 5.8–12.5) in those with HPV-negative status. There was no association of HPV status with PD-L1 expression.

The estimated median OS was 8.0 months (95% CI 6.3–10.0) in patients with oral cavity primary tumor site (*n* =143), 10.4 months (95% CI 6.9–14.9) in oropharynx (*n *=91), 12.5 months (95% CI 8.9–14.8) in larynx (*n *=137), 12.2 months (95% CI 5.7–21.0) in hypopharynx (*n* =27), and 4.0 months (95% CI 3.3–14.7) in patients with overlapping regions (*n *=11). The OS for patients with oral cavity tumors was numerically lower in the PD-L1 TC ≥ 25% population than in the PD-L1 TC < 25% population (median 6.9 months vs 9.7 months; log-rank test; *P* = 0.15). Similarly, for oropharyngeal primary site patients, those in the PD-L1 TC ≥ 25% population had a median OS of 6.3 months versus 14.8 months for patients in the PD-L1 TC < 25% population (log-rank test; *P *= 0.03) (Fig. 1d). In contrast, numerically longer survival was seen in the PD-L1 TC ≥ 25% population than in the PD-L1 TC < 25% population with hypopharyngeal primary tumors (median 21 months vs 12.2 months; log-rank test; *P* = 0.35).

**Fig. 2:**
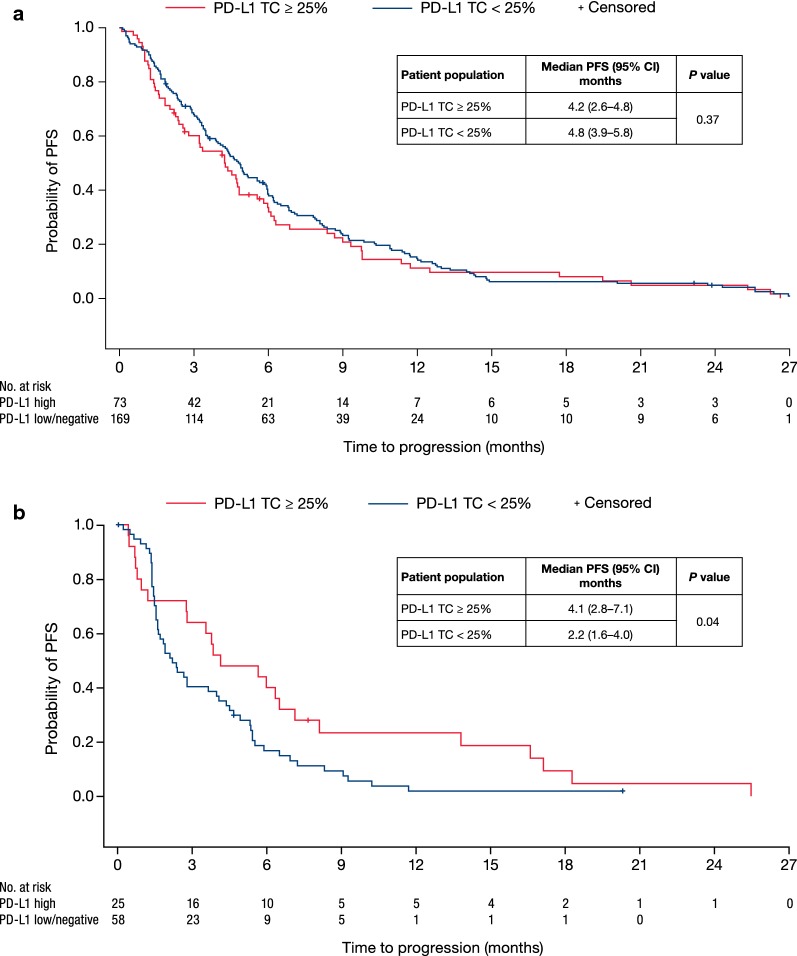
PFS by PD-L1 expression. **a** From start of first-line chemotherapy (*n* = 242) and **b** second-line chemotherapy (*n* = 83)

Median PFS from the start of first- and second-line chemotherapy was 4.6 months (95% CI 4.0–5.0) and 2.8 months (95% CI 1.9–4.4), respectively. The PFS from the start of first-line chemotherapy did not differ significantly among patients with TC ≥ 25% PD-L1 expression versus TC < 25% (median: 4.2 vs 4.8 months, *P *= 0.37) (Fig. 2a). This was similar when TC ≥ 10% PD-L1 expression versus TC < 10% and TC ≥ 50% PD-L1 expression versus TC < 50% cutoff values were applied (median 4.4 vs 4.9 months, *P* = 0.544 and median 4.8 vs 4.5 months, *P* = 0.557, respectively). However, median PFS from the start of second-line chemotherapy was significantly different between patients with TC ≥ 25% PD-L1 (*n* = 25) expression versus those with TC < 25% (*n* = 58) (4.1 months vs 2.2 months, *P *= 0.04). The difference was also significant for patients with TC ≥ 10% PD-L1 (*n* = 38) expression versus those with TC < 10% (*n* = 45) (4.1 vs 2.1 months, *P *= 0.04) and those patients with TC ≥ 50% PD-L1 (*n* = 13) expression versus those with TC < 50% (*n* = 70) (6.3 vs 2.4 months, *P* = 0.03). However, these results must be weighed against the small sample size and lack of adjustment for any confounding factors (Fig. 2b). Validation in a larger cohort of patients is required.

Among the 98 patients who had a tumor response, according to RECIST, after treatment with first-line chemotherapy, ORR was 43.9% (95% CI 33.9–54.3). Patients with PD-L1-high expressing tumors (TC ≥ 25%) had an ORR of 40.0% (95% CI 21.1–61.3, *n* = 25) and those with TC < 25% had an ORR of 44.3% (95% CI 32.4–56.7, *n* = 70) (Table 3). Among the 30 patients treated with second-line chemotherapy who had a tumor response evaluated, the ORR was 13.3% (95% CI 3.8–30.7). The ORR observed for the TC ≥ 25% cohort was 20.0% (2/10 patients; 95% CI 2.5–55.6) and those with TC < 25% had an ORR of 5.6% (1/18 patients; 95% CI 0.1–27.3) (Table 3).

**Table 3:** Response and survival by PD-L1 expression

Endpoint, *n*^a^ (%)	PD-L1 TC ≥ 25%	PD-L1 TC < 25%	PD-L1 TC ≥ 10%	PD-L1 TC < 10%	PD-L1 TC ≥ 50%	PD-L1 TC < 50%
From diagnosis date to death						
Median OS, months (range)	8.2 (6.3–10.6)	10.1 (8.3–12.2)	9.6 (7.5–12.5)	9.4 (7.9–11.4)	7.9 (5.5–12.4)	10.0 (8.5–11.8)
log-rank *P* value, PD-L1 high vs PD-L1 low/negative	0.55		0.32		0.39	
From first-line therapy						
Number evaluable	25	70				
ORR^b^, *n* (%)						
Overall response rate	10 (40.0)	31 (44.3)				
Complete response	0 (0.0)	1 (1.4)				
Partial response	10 (40.0)	30 (42.9)				
Duration of response, *n*	8	24				
Median, weeks (range)	10.6 (0.1–28.7)	15.3 (1.7–52.3)				
PFS, n	73	169	110	132	41	201
Median, months (range)	4.2 (2.6–4.8)	4.8 (3.9–5.8)	4.4 (3.3–4.9)	4.9 (3.9–6.0)	4.8 (3.2–6.1)	4.5 (3.9–5.0)
log-rank *P* value, PD-L1 high vs PD-L1 low/negative	0.37		0.54		0.56	
From second-line therapy						
Number evaluable	10	18				
ORR^b^, *n* (%)						
Overall response rate	2 (20.0)	1 (5.6)				
Complete response	0 (0.0)	0 (0.0)				
Partial response	2 (20.0)	1 (5.6)				
Duration of response, *n*	2	1				
Median, weeks (range)	10.6 (5.9–15.4)	1.3 (1.3–1.3)				
PFS, *n*	25	58	38	45	13	70
Median, months (range)	4.1 (2.8–7.1)	2.2 (1.6–4.0)	4.1 (2.2–6.5)	2.1 (1.6–3.6)	6.3 (1.2–13.8)	2.4 (1.6–3.8)
log-rank *P* value, PD-L1 high vs PD-L1 low/negative	0.04		0.04		0.03	

^a^Patients with PD-L1 result *N* = 396

^b^ORR measured by RESIST

*ORR* objective response rate, *OS* overall survival, *PD*-*L1* programmed cell death-ligand 1, *PFS* progression-free survival, RECIST Response Evaluation Criteria In Solid Tumors

### Multivariable risk factor analyses

**Fig. 3:**
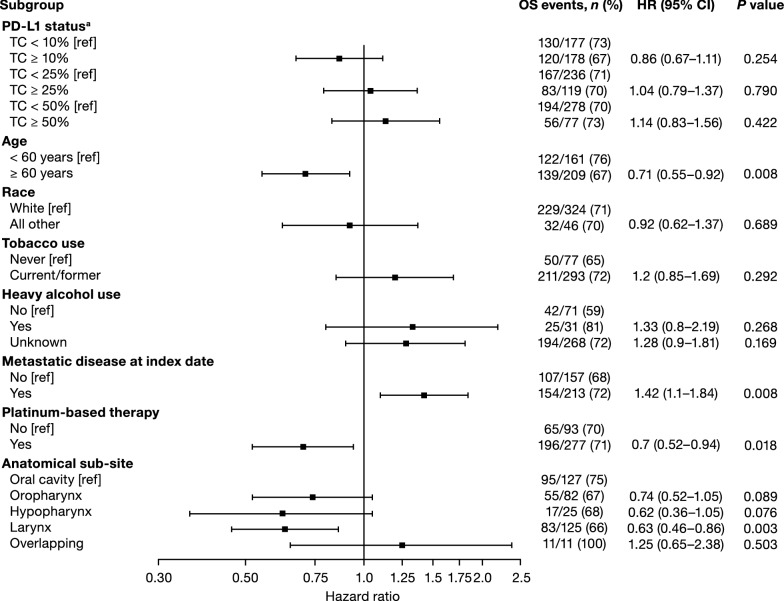
Multivariable analysis of risk factors for OS. ^a^Patients with OS data *n* = 370, patients with PD-L1 result *n* = 355

PD-L1 expression TC ≥ 25%, was not identified as a significant predictor of risk of death, with an HR of 1.04 (95% CI 0.79–1.37; *P *= 0.79), nor were cutoffs TC ≥ 10% and TC ≥ 50% (HR 0.86; 95% CI 0.67–1.11; *P *= 0.25 and HR 1.14; 95% CI 0.83–1.56; *P *= 0.42, respectively) (Fig. 3; Table 4). Metastatic disease at the time of index date was associated with increased risk of death, whereas age ≥ 60 years, platinum-based therapy, and anatomic subsite of larynx were associated with a lower risk of death regardless of the PD-L1 cutoff used (Fig. 3; Table 4).

**Table 4:** Multivariable analysis of risk factors for analyses examining PFS or OS for all-comers

Category	PFS from start of first-line therapy (*n *= 253)			PFS from start of second-line therapy (*n* = 88)			OS from index date (*n* = 370)		
	HR	95% CI	*P* value	HR	95% CI	*P* value	HR	95% CI	*P* value
PD-L1 expression high vs low^a^	1.09	(0.78–1.52)	0.63	0.58	(0.30–1.13)	0.11	1.04	(0.79–1.37)	0.790
Age < 60 vs ≥ 60 years	0.89	(0.67–1.18)	0.40	0.82	(0.45–1.47)	0.50	0.71	(0.55–0.92)	*0.008*
Race Caucasian vs other	0.59	(0.38–0.9)	0.02	0.61	(0.30–1.23)	0.16	0.92	(0.62–1.37)	0.689
Nonsmoker vs current/former smoker	0.81	(0.55–1.21)	0.31	0.97	(0.47–2.01)	0.93	1.20	(0.85–1.69)	0.292
Heavy alcohol use, no vs yes	0.97	(0.57–1.66)	0.90	1.11	(0.39–3.14)	0.85	1.33	(0.80–2.19)	0.268
Metastatic disease, no vs yes	1.27	(0.96–1.68)	0.10	0.59	(0.34–1.03)	0.06	1.42	(1.10–1.84)	*0.008*
Platinum-based therapy, no vs yes	1.10	(0.68–1.79)	0.70	3.084	(0.64–14.81)	0.16	0.70	(0.52–0.94)	*0.018*
Anatomical site vs oral cavity									
Oropharynx	0.89	(0.59–1.35)	0.58	0.88	(0.39–1.99)	0.75	0.74	(0.52–1.05)	0.089
Hypopharynx	0.65	(0.36–1.17)	0.15	0.72	(0.30–1.75)	0.47	0.62	(0.36–1.05)	0.076
Larynx	0.74	(0.51–1.06)	0.10	0.88	(0.44–1.76)	0.72	0.63	(0.46–0.86)	*0.003*
Overlapping lesion	1.05	(0.47–2.33)	0.91	0.82	(0.16–4.10)	0.81	1.25	(0.65–2.38)	0.503

Statistically significant *P* values are in italics

^a^Patients with PD-L1 result *N* = 396

*CI* confidence interval, *HR* hazard ratio, *OS* overall survival, *PD-L1* programmed cell death ligand-1, *PFS* progression-free survival

## Discussion

In this study, we investigated if PD-L1 expression was associated with survival in patients treated with standard chemotherapy.

In the entire population of this study, PD-L1 was not prognostic for survival in patients with HNSCC who received standard chemotherapy regimens. This finding was consistent with observations in randomized controlled trials of similar patients with R/M HNSCC [10, 26, 29]. In CheckMate 141, for patients treated with investigator’s choice the median OS in PD-L1 TC ≥ 1% was slightly lower than in PD-L1 TC < 1% [4.6 months (95% CI 3.8–5.8) vs 5.8 months (95% CI 4.0–9.8)] [30]. In KEYNOTE-040 the survival of patients treated with investigator's choice of standard of care (methotrexate, docetaxel, or cetuximab) did not increase with increasing PD-L1 expression [12]. Similar results have also been observed in an evaluation of commercially obtained patient samples with stage I–IV HNSCC, in which PD-L1 expression was not prognostic for OS based on a TC ≥ 25% cutoff [31].

Currently accepted prognostic markers in HNSCC include HPV status in patients with oropharyngeal carcinoma and smoking status [32]. Other researchers have identified prognostic factors including age, race, ECOG PS, prior treatments [33], C-reactive protein, leukocyte levels, and time from diagnosis to relapse [34]. In a multivariable analysis of the SUPREME-HN study we found age, platinum therapy, primary tumor location, and metastatic disease to be associated with survival. It is not surprising that metastatic disease is associated with poorer survival, this variable has been incorporated in prognostic models of survival in advanced cancers [35]. Similarly, patients healthy enough to tolerate a platinum-based therapy might be expected to survive longer. The observation here of improved survival in older patients (≥ 60 years) compared with younger patients is somewhat counterintuitive; it is generally considered that older adults have comparable survival outcomes but with increased toxicity [36]. However, a non-significantly higher survival in patients > 65 years versus < 65 years has also been shown in patients treated with investigator's choice in a retrospective analysis of CheckMate 141 [37]. In both the SUPREME-HN and the CheckMate 141 studies, investigator's choice of standard of care was used. It is possible that elderly patients were treated with taxanes, rather than cisplatin and cetuximab, due to the higher toxicities associated with the latter therapies. Later publications have indicated that docetaxel improves OS over cisplatin [38]. One could speculate that investigators selected therapies for older patients based on the toxicity profile, which were later demonstrated to be more efficacious. Urba identified race (Caucasian vs other) as prognostic for OS and PFS. In the SUPREME-HN study an association was observed that was only significant for PFS from first-line therapy; possibly because there was a smaller non-Caucasian population in this study. In a univariate analysis, Urba identified primary tumor location as negatively prognostic for survival (oral cavity vs “other”, HR 1.37, 95% CI 1.15–1.63, *P *= 0.01) and associated with reduced PFS [33]. In the multivariable analysis of the SUPREME-HN study, patients with primary tumor locations of oropharynx and hypopharynx had improved OS compared with patients with oral cavity carcinoma and survival was significantly longer in patients with tumors in laryngeal versus oral cavity sites (HR 0.63, 95% CI 0.46–0.86, *P* = 0.003). Currently smoking and HPV status are considered to be major independent prognostic factors in patients with oropharyngeal cancer [32] and recent HNSCC randomized clinical trial studies have been stratified using PD-L1 and HPV, smoking status, and performance status [39]. The SUPREME-HN study shows meaningful survival differences by primary tumor location, raising the question whether site of tumor origin should also be considered in study design and patient treatment.

The PD-L1 prevalence at TC ≥ 25% was consistent across biopsy locations: 32.1% (primary tumor), 31.8% (recurrent site), and 32.5% (metastatic site). These data suggest that any tumor lesion can be used for PD-L1 testing for HNSCC, although in this study the primary and metastatic lesions were not from the same patient. Additionally, PD-L1 expression seems to be stable across the primary versus metastatic setting, only the punch biopsy gave lower PD-L1 expression.

The prevalence of PD-L1 varied according to a number of other factors; gender (higher in females), race, region, ECOG PS 0, oral cavity cancers, and never smokers. High PD-L1 prevalence has previously been significantly associated with females, never smokers, and oral cavity in other studies of second-line patients with HNSCC [23]. The PD-L1 TC ≥ 25% prevalence varied substantially depending on the primary tumor location; from 43.5% in oral cavity to 9.5% in hypopharyngeal (see Table 1). The median OS for patients with oral cavity carcinoma was lower in PD-L1 TC ≥ 25% than PD-L1 TC < 25% patients; poor prognosis in PD-L1 TC ≥ 25% oral cavity patients has been observed by others [18]. Likewise, for oropharyngeal primary site patients, median OS in patients with PD-L1 TC ≥ 25% was less than that seen for patients with PD-L1 TC < 25% (log-rank test; *P *= 0.03; Fig. 1d). Conversely, longer survival was seen in PD-L1 TC ≥ 25% than PD-L1 TC < 25% patients with hypopharyngeal primary tumors (21 months vs 12.2 months). These data indicate that for patients with tumors of oral cavity and oropharyngeal origin, PD-L1 expression is linked to shorter survival, whereas those with PD-L1 high hypopharyngeal primary tumors live longer.

Therefore, although PD-L1 was not prognostic in the entire SUPREME-HN cohort, our data indicate PD-L1 can be both positively and negatively prognostic depending on the primary tumor location. This finding may help to explain historical conflicting views of the prognostic value of PD-L1; for example, the finding that PD-L1 expression was positively prognostic in laryngeal squamous cell carcinoma [22] but conversely associated with poor prognosis in oral squamous cell carcinoma [17].

Study limitations pertain mainly to the retrospective study design, and hence, the reliance of available information in medical charts. Quantitative analyses of risk factors were limited due to missing information on performance status, HPV status, and small sample size. This study used an assay validated for PD-L1 expression on TCs and did not investigate the prognostic value of IC PD-L1 expression. PD-L1 expression in other cellular compartments of the tumor microenvironment may be indicative of survival. The variety of scoring methods used for determining PD-L1 positivity (TCs and/or ICs) may also contribute to the apparent contradictory publications regarding its prognostic value.

Tumor stage and grade at initial diagnosis were not available for all patients since they may have received initial care in a hospital other than the investigating site. Furthermore, the definition of R/M status may have led to the exclusion of patients who received local therapies for palliative purposes, as the treatment intent was not always mentioned in the patient’s medical records. Additionally, evaluations of tumor response and progression were not evaluated via blinded, independent committee review as would be the case in clinical trials, which can lead to some variability in results. PD-L1 expression was assessed using available tissue that was not necessarily obtained at the time of initial diagnosis or at the same stage of disease for all patients. Findings from additional exploratory analyses suggest that PD-L1 expression was lower in tissue samples obtained after a patient’s prior exposure to chemotherapy than prior to initiation of chemotherapy, irrespective of tissue origin (primary tumor, recurrent site, or metastatic site). A similar finding was observed for the subset of samples from the primary tumor obtained after exposure to radiotherapy.

Since starting the SUPREME-HN study a number of immunotherapies have been approved for use in R/M HNSCC. The approvals of PD-L1 assays as companion diagnostics demonstrates the predictive nature and the value of this biomarker. As the use of immunotherapy increases the opportunity diminishes to perform a prospective study in patients treated with non-immune based treatments and thus SUPREME-HN represents a unique historical record of the prognostic value of PD-L1.

## Conclusion

There have been conflicting results reported regarding the prognostic value of PD-L1 expression on TCs. Early reports did not demonstrate any association between PD-L1 expression and OS, whereas other studies have suggested that PD-L1 expression may be associated with improved survival [17–23]. In the SUPREME-HN study, PD-L1 expression using TCs at cutoff values of 10%, 25%, and 50% was not prognostic for survival in patients with HNSCC treated with standard therapies; however, PD-L1 expression may be positively or negatively prognostic when anatomic subsites within the head and neck are considered.

In evaluating the correlation of PD-L1 and survival, previous studies did not always account for confounding factors. Based on our analysis these factors, specifically HPV status, primary tumor location, and demographic factors, may be highly relevant to OS in patients with R/M HNSCC.

## Data Availability

Data underlying the findings described in this manuscript may be obtained in accordance with AstraZeneca’s data sharing policy described at: https://astrazenecagrouptrials.pharmacm.com/ST/Submission/Disclosure.

## Abbreviations

CI: confidence interval
CT: computed tomography
ECOG: Eastern Cooperative Oncology Group
FFPE: formalin-fixed, paraffin-embedded
HNSCC: head and neck squamous cell carcinoma
HPV: human papillomavirus
HR: hazard ratio
IC: immune cell
IHC: immunohistochemistry
IO: immuno-oncology
ORR: objective response rate
OS: overall survival
PD-1: programmed cell death-1
PD-L1: programmed cell death-ligand 1
PFS: progression-free survival
PS: performance status
RECIST: Response Evaluation Criteria In Solid Tumors
R/M HNSCC: recurrent and/or metastatic head and neck squamous cell carcinoma
TC: tumor cell
